# Klotho Gene Single-Nucleotide Polymorphism and Proteinuria in a General Population

**DOI:** 10.14740/jocmr6669

**Published:** 2026-10-23

**Authors:** Masato Hamasaki, Kazuhiko Kotani, Yoshitaka Iwazu, Makoto Kuro-o

**Affiliations:** a Center for Community Medicine, Jichi Medical University, Shimotsuke-City, Tochigi 329-0498, Japan; b Department of Clinical Laboratory Medicine, Jichi Medical University, Shimotsuke-City, Tochigi 329-0498, Japan; c Division of Mineral Metabolism, Center for Molecular Medicine, Jichi Medical University, Shimotsuke-City, Tochigi 329-0498, Japan

**Keywords:** Klotho, SNP, Proteinuria, Chronic kidney disease, Genetic epidemiology, Japanese

## Abstract

**Background:**

Chronic kidney disease (CKD) is a major condition with a substantial sociomedical and economic burden. Klotho plays a role in renal protection via its antioxidant and anti-inflammatory properties. Proteinuria is an early indicator of CKD. Although experimental studies have demonstrated the biological effects of Klotho in CKD, its clinical relevance to proteinuria remains unclear.

**Methods:**

The present study investigated the association between the A/T alleles of the Klotho gene rs650439 single-nucleotide polymorphism (SNP) and proteinuria in a general Japanese population. Clinical and genotyping data from a total of 916 community-dwelling participants (mean age = 63 years old) were analyzed.

**Results:**

Participants carrying the minor TT genotype accounted for 13.2% of the population. The prevalence of proteinuria was significantly higher in the TT group than in the AA + AT group (15.7% vs. 7.3%, P < 0.01). Logistic regression analysis showed that the TT genotype was significantly associated with proteinuria (crude odds ratio (OR) = 2.37, 95% confidence interval (CI) 1.35–4.14). This association remained significant after adjustment for age, sex, and cardiometabolic factors (adjusted OR = 2.07, 95% CI 1.14–3.77).

**Conclusions:**

These findings suggest that the TT genotype of Klotho gene rs650439 SNP may serve as a genetic marker for early renal proteinuria. Further research is required to confirm the findings.

## Introduction

Alpha-Klotho (α-Klotho) was initially identified as an anti-aging gene, as genetic disruption of this locus results in a phenotype resembling advancing aging [1]. Transgenic overexpression of α-Klotho improves disorders associated with α-Klotho deficiency and significantly prolongs lifespan in murine models [1, 2]. α-Klotho shows pleiotropic effects across multiple organ systems and participates in a wide range of physiological processes [3, 4]. Structurally, α-Klotho is a type I single-pass transmembrane protein characterized by an extracellular domain containing β-glucosidase–like motifs, as referred to as Kl domains [5]. The expression of α-Klotho is limited to several tissues [1], such as the kidney, parathyroid glands, and sinoatrial nodes, where it forms tetrameric complexes with fibroblast growth factor (FGF) receptor 1c (FGFR1c), FGFR3c, or FGFR4. In this configuration, α-Klotho acts as an essential high-affinity co-receptor for circulating FGF23, thereby regulating systemic mineral metabolism [4, 6]. In the kidney, α-Klotho mediates diverse biological functions, including suppression of the sodium-dependent phosphate cotransporter (NaPi) transporter activity in proximal tubules and protection against oxidative stress and inflammatory injury.

Experimental studies have demonstrated that α-Klotho is predominantly expressed in renal tubular epithelial cells and podocytes, where it protects the kidney from ischemic, toxic, and metabolic injury [7]. Furthermore, α-Klotho deficiency leads to renal fibrosis, vascular calcification, and premature aging phenotypes in mice [8, 9], highlighting its critical role in maintaining renal and vascular homeostasis. The extracellular domain of α-Klotho is shed from the cell surface through proteolytic cleavage [4] and can be detected in circulation, urine, and cerebrospinal fluid [5]. Among peripheral tissues, the kidney exhibits the highest level of α-Klotho expression, and substantial evidence indicates that the kidney is the principal source of circulating soluble α-Klotho [3]. Soluble α-Klotho can form a ternary complex with FGF and FGFR, thereby functioning as a co-receptor. However, accumulating data suggest that soluble α-Klotho predominantly acts in an FGF23- and FGFR-independent manner after entering the circulation. Functionally, soluble α-Klotho mediates a broad spectrum of biological effects, including antioxidant and anti-senescent activities, promotion of autophagy, inhibition of apoptosis and fibrosis, support of stem cell function, and modulation of insulin signaling [10]. In human studies, reduced circulating α-Klotho levels have been associated with chronic kidney disease (CKD), hypertension, and cardiovascular events [11].

CKD represents a major clinical manifestation of renal damage and is characterized by persistent structural and/or functional abnormalities of the kidney lasting for more than 3 months [12]. CKD is clinically defined by a sustained reduction in glomerular filtration rate (GFR) and/or evidence of kidney damage, including albuminuria or imaging abnormalities [12]. Epidemiological studies indicate that CKD is prevalent worldwide, affecting approximately 10–15% of the adult population [13, 14]. CKD is a progressive disorder, and continued decline in renal function can ultimately lead to end-stage kidney disease, which requires renal replacement therapy, such as hemodialysis, peritoneal dialysis, or kidney transplantation [15]. In addition to progression to dialysis dependence, CKD is an independent risk factor for cardiovascular disease and all-cause mortality [16].

Proteinuria is a well-recognized indicator of renal damage and an established predictor of cardiovascular disease morbidity and mortality, independent of GFR [17]. It represents an early manifestation of impaired glomerular filtration resulting from podocyte and glomerular dysfunction [18]. The incidence of proteinuria increases with age, hypertension, and diabetes mellitus [18]. Therefore, these populations should be screened for proteinuria to prevent CKD progression [12]. Although experimental studies have shown that α-Klotho deficiency promotes podocyte and glomerular injury [19], the relationship between Klotho and proteinuria has not been fully elucidated in the general population.

The Klotho gene single-nucleotide polymorphism (SNP) rs650439 has been associated with an increased risk of vascular disorders and renal dysfunction [20, 21]; however, these studies focused on advanced renal disease rather than the proteinuria phase. Therefore, the present study aimed to investigate the association between Klotho rs650439 and proteinuria in a general Japanese population.

## Materials and Methods

This study was conducted in the health screening setting for community-dwelling residents. The study was approved by the Ethics Committee of Jichi Medical University, and conducted in accordance with the Declaration of Helsinki, and approved by the Ethics Committee of Jichi Medical University (No. 19-023). All participants provided written informed consent. Clinical data from a total of 916 participants, including information on demographics, lifestyles, laboratory indices, and the presence or absence of hypertension and diabetes mellitus, were used. Information on demographics, lifestyles, and medical history was obtained by questionnaire. Hypertension is defined as a systolic blood pressure of 140 mm Hg and/or a diastolic blood pressure of 90 mm Hg [22] or a history of hypertension and/or the prescription of antihypertensive drugs. Diabetes mellitus is defined as fasting blood glucose of 126 mg/dL or random blood glucose of 200 mg/dL [23] or a history of diabetes mellitus and/or the prescription of antidiabetic drugs. Participants with a history of cardiovascular and/or cerebrovascular diseases and/or apparent renal disorders were excluded because of the purpose of primary prevention in this study.

Proteinuria was assessed using a single dipstick measurement of a spot urine sample obtained at the time of the health screening [24]. Urinary protein was semi-quantitatively examined using a standard urine reagent strip method according to the manufacturer’s instructions. The results were recorded as negative (−), trace (±), 1+, 2+, 3+, or 4+ based on colorimetric changes. Proteinuria was defined as a dipstick result of 1+ or greater of 1+. The estimated glomerular filtration rate (eGFR) was calculated based on the value of serum creatinine [25].

Genotyping of the Klotho gene rs650439 SNP was performed using a custom TaqMan SNP Genotyping Assay (Thermo Fisher Scientific, CA, USA). Genomic DNA was extracted from peripheral blood samples using a standard method [26]. The assay was designed with allele-specific probes, and polymerase chain reaction (PCR) amplification was conducted on a StepOnePlus Real-Time PCR System (Thermo Fisher Scientific) according to the manufacturer’s instructions. Allele calling was automatically performed using StepOne Software v2.3.

Data were analyzed for the rs650439 allele. The TT genotype is the minor genotype. The participants with the TT genotype were then compared with those carrying the combined AA + AT genotypes (recessive model) according to the previous reports [20]. Continuous variables are expressed as the mean ± standard deviation (SD). Comparisons among groups were conducted using Student’s *t*-test. Categorical variables are presented as numbers (%) and were compared using the χ^2^ test. Genotype frequency was tested for Hardy–Weinberg equilibrium using the χ^2^ test. Logistic regression analyses were performed to evaluate the association between rs650439 SNP and proteinuria by calculating crude and adjusted odds ratios (ORs) with 95% confidence intervals (CIs). The dependent variable was the presence of proteinuria. The independent variables as an adjusted factors for genotype were age, sex, eGFR, hypertension, and diabetes mellitus in model 1 and the variables in model 1 plus alcohol consumption, smoking, body mass index (BMI), and total cholesterol in model 2. Statistical significance was set at P < 0.05. All statistical analyses were performed using Python version 3.11 with the Pandas, NumPy, SciPy, and Statsmodels libraries.

## Results

Overall, 121 (13.2%) patients carried the TT genotype of Klotho gene rs650439 SNP, whereas 795 (86.8%) carried the AA (n = 430) or AT (n = 365) genotypes. The allele distribution of Klotho gene rs650439 SNP in the study population was in accordance with the Hardy–Weinberg equilibrium (P = 0.831). We observed no significant differences between the TT and AA + AT groups with respect to age, sex, alcohol consumption, current smoking, BMI, total cholesterol levels, hypertension, or diabetes mellitus (Table 1). The prevalence of proteinuria was significantly higher in the TT group than in the AA + AT group (15.7% vs. 7.3%, P < 0.01). 18.3% (n = 168) had an eGFR < 60 mL/min/1.73 m^2^, which is suggestive of CKD [24]. The population with such a low eGFR or higher serum creatinine level was higher in the TT group than in the AA + AT group.

**Table 1:** Characteristics According to the Klotho Gene rs650439 SNP Alleles

Variable	All (n = 916)	AA + AT (n = 795)	TT (n = 121)	P-value
Age, years	63 ± 14	63 ± 14	65 ± 12	0.13
Male, n (%)	415 (45.3%)	357 (44.9%)	58 (47.9%)	0.60
Alcohol consumption, n (%)	437 (47.7%)	378 (47.5%)	59 (48.8%)	0.88
Current smoking, n (%)	327 (35.7%)	285 (35.8%)	42 (34.7%)	0.89
BMI, kg/m^2^	22.9 ± 3.2	22.8 ± 3.3	23.2 ± 3.1	0.59
Total cholesterol, mg/dL	205.9 ± 35.0	206.3 ± 35.0	203.7 ± 35.3	0.75
Creatinine, mg/dL	0.74 ± 0.19	0.73 ± 0.18	0.78 ± 0.23	0.04*
eGFR, mL/min/1.73 m^2^	74.9 ± 15.3	75.2 ± 15.0	72.5 ± 16.7	0.19
eGFR, < 60 mL/min/1.73 m^2^	168 (18.3%)	136 (17.1%)	32 (26.4%)	0.02*
Hypertension, n (%)	385 (42.0%)	328 (41.3%)	57 (47.1%)	0.26
Diabetes mellitus, n (%)	92 (10.0%)	79 (9.9%)	13 (10.7%)	0.91
Proteinuria, n (%)	77 (8.4%)	58 (7.3%)	19 (15.7%)	< 0.01*

Values are shown as the mean ± standard deviation for continuous variables and as numbers (%) for categorical variables. Genotypes were coded as TT versus AA + AT. Student’s *t*-test was used for continuous variables, whereas the chi-square test was used for categorical variables. An asterisk (*) indicates statistical significance (P < 0.05). BMI: body mass index; eGFR: estimated glomerular filtration rate.

In univariate analysis, the TT genotype was significantly associated with proteinuria (OR = 2.37, 95% CI 1.35–4.14, P < 0.01). This association remained significant after adjustment for model 1 (adjusted OR (aOR) = 2.08, 95% CI 1.15–3.77, P = 0.02) and model 2 (adjusted OR (aOR) = 2.07, 95% CI 1.14–3.77, P = 0.02). In addition, eGFR (aOR = 0.98, 95% CI 0.96–0.99, P = 0.01), hypertension (aOR = 2.02, 95% CI 1.16–3.50, P = 0.01), and diabetes mellitus (aOR = 3.28, 95% CI 1.80–5.97, P < 0.01) were independently associated with proteinuria (Table 2).

**Table 2:** Univariate and Multivariate Logistic Regression Analysis of Proteinuria

Variable	Crude OR (95% CI)	P-value	Adjusted OR (95% CI) of model 1	P-value	Adjusted OR (95% CI) of model 2	P-value
Age, years	1.03 (1.01–1.05)	< 0.01*	1.01 (0.99–1.03)	0.32	1.01 (0.99–1.03)	0.37
Male	2.05 (1.27–3.30)	< 0.01*	1.83 (1.11–3.02)	0.02*	1.47 (0.75–2.88)	0.26
Alcohol consumption	1.27 (0.80–2.03)	0.31	-	-	1.15 (0.66–2.01)	0.62
Current smoking	1.56 (0.98–2.50)	0.06	-	-	1.13 (0.60–2.15)	0.71
BMI, kg/m^2^	1.03 (0.97–1.10)	0.30	-	-	1.00 (0.93–1.08)	0.95
Total cholesterol, mg/dL	0.99 (0.98–1.00)	0.01*	-	-	0.99 (0.99–1.00)	0.06
eGFR, mL/min/1.73 m^2^	0.97 (0.96–0.99)	< 0.01*	0.98 (0.96–1.00)	0.01*	0.98 (0.96–0.99)	0.01*
Hypertension	2.97 (1.81–4.85)	< 0.01*	2.07 (1.21–3.55)	< 0.01*	2.02 (1.16–3.50)	0.01*
Diabetes mellitus	4.39 (2.53–7.63)	< 0.01*	3.29 (1.82–5.94)	< 0.01*	3.28 (1.80–5.97)	< 0.01*
TT genotype (vs. AA + AT)	2.37 (1.35–4.14)	< 0.01*	2.08 (1.15–3.77)	0.02*	2.07 (1.14–3.77)	0.02*

Crude odds ratios (ORs) were calculated using univariate logistic regression of proteinuria with TT genotype. Adjusted ORs were calculated using multivariate logistic regression of proteinuria with TT genotype in model 1 (to which age, sex, eGFR, hypertension, diabetes mellitus, and genotypes were entered) and model 2 (to which age, sex, alcohol consumption, smoking, BMI, total cholesterol, eGFR, hypertension, diabetes mellitus, and genotypes were entered). Genotypes were coded as TT versus AA + AT. An asterisk (*) indicates statistical significance (P < 0.05). BMI: body mass index; eGFR: estimated glomerular filtration rate.

## Discussion

The present study demonstrated a significant association between the minor TT genotype of the Klotho rs650439 SNP and the presence of proteinuria in a general population, and their association remained significant even after adjusting for confounders. Proteinuria is a recognized marker of early CKD development [27–30]. Therefore, this variant may have potential utility as a genetic marker for identifying individuals at increased risk of CKD and for guiding early preventive management strategies.

α-Klotho contributes to renal protection by suppressing podocyte apoptosis, inhibiting the transforming growth factor-β/Smad signaling pathway involved in cellular differentiation, and reducing oxidative stress and inflammatory cytokine expression [31]. Low α-Klotho levels have been implicated in the early stages of renal injury, especially in high-risk populations [32, 33]. To date, individuals carrying the minor TT genotype have been reported to exhibit lower α-Klotho levels than those carrying AA or AT alleles [20]. These findings may imply a biological association between Klotho rs650439 and proteinuria in relation to α-Klotho levels, despite the present study being limited to only an observational genetic association.

If the association between Klotho rs650439 and proteinuria is mediated by circulating soluble α-Klotho levels, genotype-based stratification may also have therapeutic implications. Supplementation with soluble α-Klotho has shown reno-protective effects in animal models, particularly in the early stages of renal injury [34–39]. Moreover, in a clinical study, treatment with sodium–glucose cotransporter 2 inhibitors or renin-angiotensin system blockers in patients with diabetic nephropathy is associated with reduced proteinuria and increased circulating soluble α-Klotho levels [40]. Future studies will be necessary for genotype-based therapy.

Proteinuria is a predictor of cardiovascular disease mortality and decline in GFR than GFR itself [41]. However, the pathophysiological mechanisms linking albuminuria to cardiovascular disease are of interest but have not been fully elucidated. One potential mechanistic link is disordered phosphate metabolism. Elevated plasma phosphate concentrations, even within the upper limits of the normal range, are associated with an increased risk of cardiovascular events and vascular calcification, independent of renal function [42, 43]. Clinically, patients with higher serum phosphate levels tend to exhibit more severe proteinuria, elevated circulating FGF23 concentrations, and reduced soluble α-Klotho levels, which may be associated with altered FGF23/Klotho signaling [8, 44]. Although serum and urinary phosphorus levels and serum FGF23 levels were not examined in the present study, phosphate metabolism is of concern in the association between the Klotho SNPs and proteinuria. Further investigation of this point is required.

In addition, the TT genotype of the Klotho rs650439 has been reported to be associated with an increased incidence of stroke in hypertensive patients [20]. This may be mediated by the possible effect of the TT genotype on circulating soluble α-Klotho levels, as shown in Figure 1. In the overall views, a mechanistic link between proteinuria, disruption of the renal FGF23/Klotho axis, and disordered phosphate metabolism is presumable, ultimately contributing to the development of cardiovascular disease and progressive renal dysfunction.

**Figure 1:**
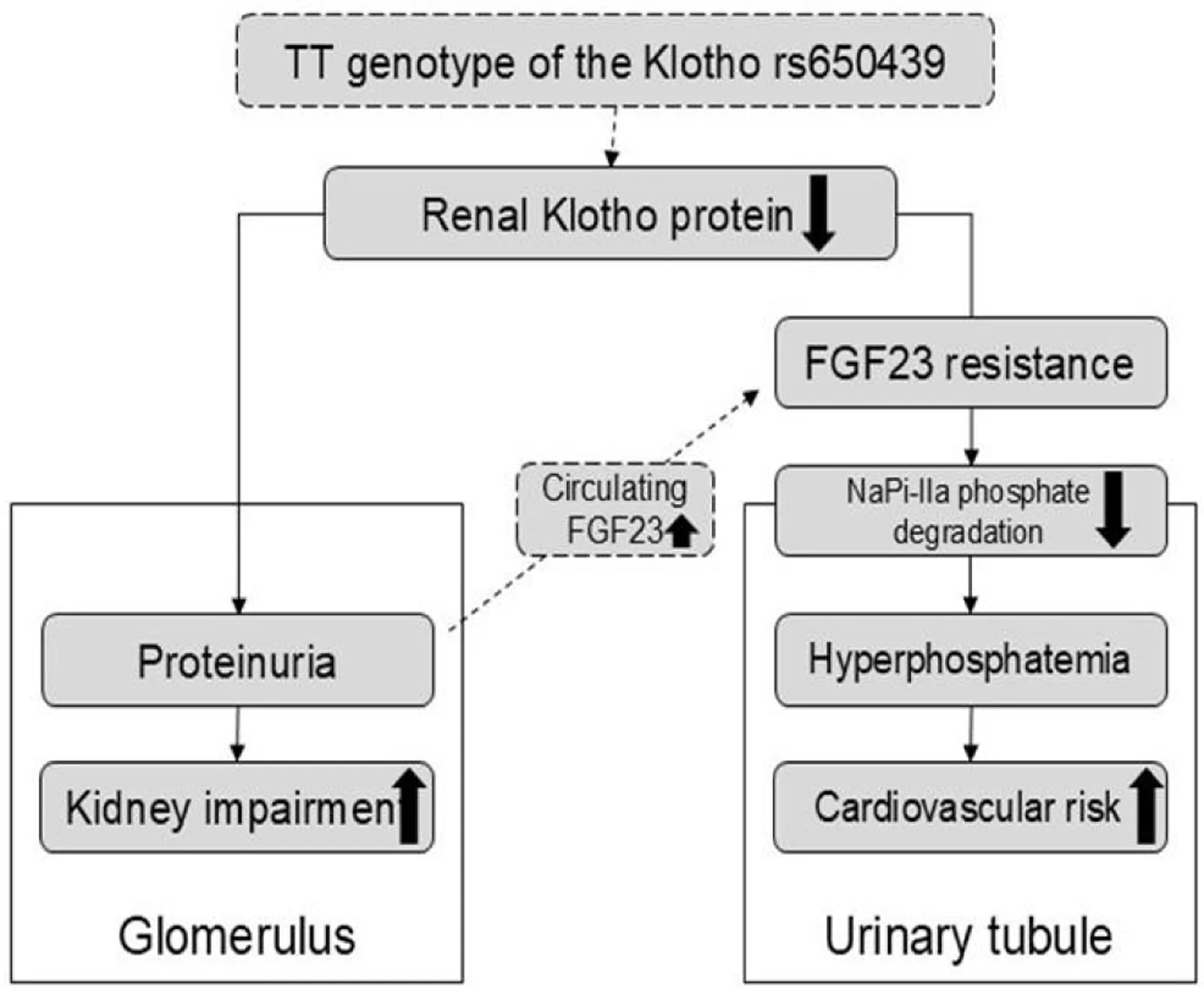
Possible mechanistical linking proteinuria, disordered phosphate metabolism, and cardiovascular risk. Proteinuria downregulates renal Klotho expression, impairing FGF23 signaling and NaPi-IIa degradation in the proximal tubule. This leads to reduced urinary phosphate excretion, phosphate retention, and vascular toxicity, thereby linking albuminuria to cardiovascular risk and progressive renal dysfunction. Solid arrows indicate established relationships, whereas dashed arrows indicate modulatory or proposed relationships. FGF23: fibroblast growth factor 23; NaPi-IIa: sodium-dependent phosphate cotransporter IIa.

In the present study, hypertension and diabetes mellitus are respectively associated with proteinuria. Hypertension and diabetes mellitus have been already reported to be established risk factors for proteinuria [45]. The findings of the present study are thus consistent with this existing knowledge [45]. In hypertension, an elevated glomerular pressure leads to impair the filtration membrane, causing proteins such as albumin to leak into the urine [46]. In diabetes mellitus, chronic hyperglycemia can lead to glycation and thickening of the glomerular basement membrane, increasing its permeability and resulting in proteinuria [47].

The present study had several limitations. First, the cross-sectional design precludes any causal inferences. Second, the levels of circulating α-Klotho, albumin-to-creatinine ratio, and/or 24-h proteinuria were not measured though these might provide additional knowledge to confirm the biological association between the TT genotype and proteinuria. Third, although dipstick is a standard method for detecting proteinuria in health screening settings, its sensitivity/specificity is limited in defining precise categorization of kidney disease. Fourth, the severity of hypertension and diabetes mellitus was not reflected in the analysis as there was not sufficiently such information. Fifth, we focused on rs650439 because the association with α-Klotho levels has been reported before [20], but the other SNPs might be examined. Future investigations are expected to confirm the results of the present study and to clarify the mechanisms underlying these findings.

### Conclusions

In conclusion, the present study showed a significant association between the minor TT genotype of Klotho gene rs650439 SNP and the presence of proteinuria in a general Japanese population. These findings suggest that this variant may be a genetic marker of proteinuria in the early stage of CKD. Further studies are required to confirm this association and to clarify its clinical relevance.

## Data Availability

The data for the study are available upon reasonable reasons by the corresponding author with ethics committee regulations.

## Abbreviations

α-Klotho: alpha-Klotho
BMI: body mass index
CI: confidence interval
CKD: chronic kidney disease
eGFR: estimated glomerular filtration rate
FGF: fibroblast growth factor
FGFR: fibroblast growth factor receptor
GFR: glomerular filtration rate
NaPi-IIa: sodium-dependent phosphate cotransporter IIa
OR: odds ratio
SD: standard deviation
SNP: single-nucleotide polymorphism
